# Biomedical Big Data: New Models of Control Over Access, Use and Governance

**DOI:** 10.1007/s11673-017-9809-6

**Published:** 2017-10-05

**Authors:** Effy Vayena, Alessandro Blasimme

**Affiliations:** Health Ethics and Policy Lab—Department of Health Sciences and Technology, ETH Zurich, Auf der Mauer, 17, 8001 Zurich, Switzerland

**Keywords:** Big Data, Control, Ethics, Privacy, Informed consent, Governance

## Abstract

Empirical evidence suggests that while people hold the capacity to control their data in high regard, they increasingly experience a loss of control over their data in the online world. The capacity to exert control over the generation and flow of personal information is a fundamental premise to important values such as autonomy, privacy, and trust. In healthcare and clinical research this capacity is generally achieved indirectly, by agreeing to specific conditions of informational exposure. Such conditions can be openly stated in informed consent documents or be implicit in the norms of confidentiality that govern the relationships of patients and healthcare professionals. However, with medicine becoming a data-intense enterprise, informed consent and medical confidentiality, as mechanisms of control, are put under pressure. In this paper we explore emerging models of informational control in data-intense healthcare and clinical research, which can compensate for the limitations of currently available instruments. More specifically, we discuss three approaches that hold promise in increasing individual control: the emergence of data portability rights as means to control data access, new mechanisms of informed consent as tools to control data use, and finally, new participatory governance schemes that allow individuals to control their data through direct involvement in data governance. We conclude by suggesting that, despite the impression that biomedical big data diminish individual control, the synergistic effect of new data management models can in fact improve it.

## 1. Introduction

Large reams of data are generated, collected, and processed each day for an indefinite variety of purposes. A portion of these data is the output of scientific experiments in all fields of human knowledge, from astronomy to sociology and from climate science to physics. However, a great deal of data stems from the activities of social infrastructures, like the state’s administration, financial systems, telecommunications networks, civil aviation, or the Internet. In this respect, a vast part of the world’s data comprises information that keeps track of what individuals do in the course of their daily life and activities.

The capacity to exert control over the circulation of such personal and professional information is a fundamental premise to crucial values such as autonomy, privacy, and trust. For the purposes of the present paper, we will speak of personal information in the domain of individual health. To this aim, we define personal health information as all information about the health state of an identifiable individual (at any time) obtained through the analysis of health data. Personal health information can either be acquired through data collected by direct observation or measurement of individuals’ behaviour, bodily traits, and pathophysiological state; or be inferred through the analysis of other types of data. We discuss such expanded interpretation of health-related data at length in the next section.

Individual control over personal health information in healthcare and clinical research is generally achieved indirectly. Instead of having granular control over the circulation of such information, patients and research participants generally agree to specific conditions of exposure. Such conditions can be openly stated in informed consent documents or be implicit in the norms of confidentiality that govern the relationship between patients and healthcare professionals. And yet, as medicine—like so many other activities—becomes a data-intense enterprise, informed consent and medical confidentiality seem to offer only limited amounts of control over the production, collection, use, and circulation of health-relevant data. Empirical evidence suggests that while people hold the capacity to control their data in high regard, they experience a sense of loss of such control in the online environment. According to the latest Eurobarometer survey, only 15 per cent of those who provide personal information online feel they have complete control over their personal data.^1^ All other respondents think they have either only partial control (50 per cent) or no control at all (31 per cent) over what is done with their personal data. Moreover, 69 per cent of respondents believe that express permission should be asked before collecting and using their personal data and about the same percentage claims to be concerned about their personal data being used for purposes they had not initially authorized. Along similar lines, according to a recent survey on Americans’ attitudes towards privacy, security, and surveillance, 93 per cent of respondents agreed that being in control of who can get information about them is important or very important (Madden and Rainie 2015).

These data refer to the entire online environment and not specifically to the health sector. In fact, at least in the European study, when people were asked about which types of institutions they trust most in handling their data, health and medical institutions ranked on top. However, as we argue in this paper, within the evolving health data ecosystem, data coming from different contexts (e.g. from research rather than from clinical care) are not clearly demarcated from each other. This poses significant challenges to both the control of personal data uses and subsequently to the trustworthiness of the users.

In what follows, we provide an expanded account of health data and define the normative importance of data control along with the challenges it raises (sections 2 and 3). We then explore novel models of informational control in data-intense healthcare and clinical research which aim to enhance individual control of data (section 4). More specifically, we discuss the emergence of data portability rights as means to control data access; new models of informed consent and data transactions as tools to control data use; and finally, new participatory schemes providing individuals with the option to control their data through direct involvement in data governance. We conclude by suggesting that, despite the impression that biomedical big data diminish individual control, the synergistic effect of new data management models can in fact improve it.

## 2. The Evolving Nature of Health Data

Health data conventionally include laboratory test results, diagnostic images, medical records, public health registry data, and data produced in the context of biomedical or clinical research. However, the notion of health data has expanded considerably in the last two decades (Vayena and Gasser 2016). For instance, the ever decreasing cost of genome sequencing has spurred the generation—for both clinical and research purposes—of unprecedented amounts of genomic data (Stephens et al. 2015), one of the most discussed novel types of health data. Owing to technological progress, individual medical records ever more frequently come grouped in the form of comprehensive electronic health records—a shift in format that is enabling previously unthinkable uses of patients’ data (Jensen, Jensen, and Brunak 2012; Coorevits et al. 2013). Altogether new forms of data are also making their way into medicine and medical research, including data produced outside the perimeter of clinical care and medical or clinical research.

The term “biomedical big data” designates all health-relevant data that can be made interoperable and thus amenable to predictive data mining for health-related purposes. Biomedical big data range from data generated by health services, public health activities, and biomedical research, to data registering exposure to environmental factors like sunlight or pollution, or data revealing lifestyle, socioeconomic conditions, and behavioural patterns, such as data from wellness and fitness applications, social media, and wearable devices (Vayena, Dzenowagis, and Langfeld 2016). Biomedical big data contain detailed information about people’s characteristics at phenotypic, genotypic, behavioural, and environmental levels. Thanks to the use of modern data mining and deep-learning techniques, this type of data can prove extremely valuable to making health-related predictions for both individuals and populations (Freifeld et al. 2014; Executive Office of the President, President’s Council of Advisors on Science and Technology 2014; Krumholz 2014; Khoury and Ioannidis 2014: Bender 2015; Hill 2012).

For this reason, biomedical big data form what has been labelled a “data ecosystem”—an analogy that stresses the interdependence of the actors and processes that rely on the production and circulation of data as a key resource for their respective activities (Vayena and Gasser 2016). The idea of a data ecosystem highlights two important features of biomedical big data: first, the fact that they blur conventional distinctions between data types produced in different settings, thus turning virtually any form of data into health-relevant data (Jain et al. 2015; Hawgood et al. 2015); and second, that data governance will likely need to draw on a wider array of relevant stakeholders that should encompass actors well beyond the biomedical community, including, primarily, data subjects.

## 3. Does Data Control Matter?

Informational control amounts to the power to decide on the conditions of exposure of health data and personal health information.^2^ For instance, one can be said to have control over such information when one is able to decide whether that information can be acquired by another subject (for example a pharmaceutical company), shared with other researchers, or used for previously unanticipated purposes.

Controlling big biomedical data that refer to ourselves can be connected to other valued states, to which control is—one way or another—germane or instrumental. And yet, we are not implying that individual control is all that matters in the protection and promotion of people’s interests concerning health data and personal health information. Beyond individual control, legislative provisions, oversight mechanisms, and procedures for the use of health data are crucial to ensure that those interests are respected.

Data control should not be understood as an absolute value but rather as a precondition to a host of valued states including autonomy, self-determination, privacy, trust, transparency, and accountability. Such values are held in high regard in any domain of human activity, but they acquire even greater importance in the context of healthcare and health-related research, given the vulnerability of patients and research participants. It will therefore be useful to spell out the importance of control within the articulated set of values and interests that it is supposed to serve. For starters, as autonomous individuals, we have an interest in being in charge of decisions that affect us personally. Decisions about data that describe who we are and how we live do indeed concern us directly. Not being able to make those decisions can thus undermine our autonomy. Controlling the flow of information about ourselves is instrumental to our personal development as it enables us to present ourselves in different ways to different people and in different social contexts (Rachels 1975; Parent 1983). This is especially relevant in the case of potentially stigmatizing medical conditions. Control of personal data is also important for one’s right to privacy. Although not a sufficient condition of privacy (Allen 1999; Solove 2012), control is certainly crucial to keep personal information in a state of restricted access and to avoid the sense of threat that comes with the mere risk of privacy intrusions (Westin 1967; Parker 1973). Given the harmful consequences of privacy breaches in the case of personal health information (for example in terms of insurance coverage discrimination), the value of privacy in the healthcare sector hardly needs to be stressed any further.

Data control on the part of data subjects is also conducive to transparency, accountability, and trust, as it requires data holders to inform data subjects about current uses of their data. Accordingly, they can decide whether or not to keep their data available. In the health context, such mechanisms are considered the baseline for building and maintaining trust in healthcare and research institutions. This also demands that an organization clearly identifies and discloses who is accountable for data management, for secure storage and access procedures, as well as for monitoring the use of such data. Those mechanisms, in turn, encourage people to make their personal data available in the first place under a governance framework that they trust, thus making data analysis possible for a number of socially useful activities—including the improvement of healthcare provision and the advancement of biological and medical sciences.

Other than catering to important values and interests, however, playing an active role in the management of one’s own personal data can be a hallmark of a special type of character—one that shows a disposition to prudence, modesty, and self-reliance. This might explain why we are used to thinking about control mainly as a *direct* individual responsibility. But if one considers the exponential growth in the amount of data being generated through digital technologies, then framing control as a personal responsibility alone might result in an excessive burden for individuals. Faced with the onerous responsibility of controlling what happens to the multiplicity of their data, people might eventually prefer to eschew activities that entail data collection (Brunton and Nissenbaum 2016). This would be unfortunate in the case of those data-driven activities—like health research based on biomedical big data—from which public utility is legitimately expected.

Another risk relates to what we can call “the illusion of control” (Brandimarte et al. 2009)—namely the false sense of control generated through the individuals’ required agreements to terms of service, which, as evidence repeatedly shows, individuals do not read or understand (Solove 2012). It should be noted, however, that one possible way of using direct control over one’s data is to cede control to another party, as is often the case in the healthcare sector. In this case, the capacity to exert control tends to undermine the very ideals of self-directedness that control is supposed to serve. As a consequence, understanding control merely as a personal responsibility can lead towards sub-optimal outcomes in terms of autonomy and self-determination. Despite this potential contradiction, ever more frequently, claims to health data control are coming precisely from people who are interested in releasing their personal data in the public sphere.

The OpenSNP platform, for example, is designed to allow people to take genomic data obtained through direct-to-consumer genetic testing companies and to release them on an unrestricted website with no privacy guarantee whatsoever (Haeusermann et al. 2017). Therefore, the right to informational self-determination (that is, the right to *control* what personal information is disclosed when, to whom, and for what purpose) should not be understood as a means to prevent data collection and data use. Nor should it be interpreted as a potential threat to the benefits that such activities may contribute to in the case of healthcare and health-related research. Improved health services, more effective drugs, better public health interventions, and progress in basic science are among the expected benefits of data-driven medicine. The value of these developments is self-evident. Yet, individual control can be used in ways that do not promote such outcomes. Nevertheless, in the absence of sufficient evidence, it is premature to think that people exercising data control rights will preferably make highly restrictive uses of those rights and end up hindering progress in healthcare. In fact, extreme cases of data control, such as OpenSNP, show that the opposite may be the case. What is more, the availability of data control—being a sign of respect for people’s interests—may promote rather than hinder the propensity to share data for health and health research related purposes. Finally, there is an important counter-incentive to using data control to restrict data availability: since one of the promises of data-intense medicine is the provision of personal health information to health data contributors, people who adopt an overly self-protective posture will exclude themselves from the benefit of receiving clinically relevant information about their health.

Informational control also bears on another typically individual entitlement, namely ownership. It is generally assumed that one should have control over what is hers or, otherwise stated, that owning entails the privilege of exerting some exclusive form of *direct* control over what is owned. These intuitions establish a conceptual link between control and ownership. Moreover, some scholars have famously argued that informational privacy boils down to a form of control over informational resources that people *own* (Thomson 1975). And yet, establishing who owns biomedical big data is notoriously difficult. With health-related data for instance, it is not generally the case that clinical or research data are the property of data subjects in any meaningful sense. Clinical trials data, for instance, are the property of the sponsoring company; and research data collected through public funding are not treated as the property of either the research participants or the researchers. Nonetheless, both participants and researchers have some rights on data that could be associated with property. Research participants, for instance, can require the destruction of their data. And research institutions can sell or even share their data for free—but generally only if donors have initially consented to that. With biomedical big data, establishing property rights is further exacerbated by the convergence of data produced in clinical, academic, and commercial settings and data retrieved from online environments.

Nonetheless, some have argued in favour of full-blown property rights on personal data as a means to reinforce privacy (Westin 1967, 324–325).This type of framing, however, has generated criticism for both personal data in general (Litman 2000) and for personal health data in particular (Evans 2011). A classic objection stems from the consideration that in Western legal systems property rights can be violated under special circumstances and thus would not fully protect against unauthorized access to even privately owned data. Moreover, data property implies the possibility of selling one’s data to another party, but this, in turn, implies losing control over such data for good. In light of these considerations, it seems that framing control primarily as a direct responsibility of the data subject, or considering it as an emanation of her proprietary rights on her data, might create more problems than it actually solves. In this respect, we concur with claims about the illusionary nature of full individual data control as a means to promote data subjects’ interests in the era of big data (Solove 2012). Yet, we set out to show that, at least in the field of biomedical big data, mechanisms are evolving that could give research participants more control.

### 3.1 Typical Means of Control Over Health Data

In healthcare and health research, often data subjects are either healthy or diseased volunteers who enrol in clinical studies or, alternatively, individuals who donate samples and health-relevant data irrespective of their health status. The way in which these data subjects can exert control over their data, however, is mainly indirect. For instance, procedures like informed consent—a keystone of medical ethics—might disclose information to data subjects as to how their data and information will be used, stored, distributed, and protected from unauthorized access. Informed consent documents explain what are the conditions of exposure for health data and personal health information in a given study. It thus allows participants to decide whether or not those conditions correspond to their expectations and best interests. However, there is quite some variability as to the level of detail regarding data exposure in different types of consent forms. For example, research participants who volunteer to provide samples and data for the constitution of a research biobank cannot know in advance who will access their data in the future and for which scientific purposes. In those circumstances, data subjects generally sign broad consent forms, that is, consent forms that do not specify the conditions of data exposure in any great detail but nonetheless set some limitations to the use of the donated resources (Grady et al. 2015). This model has attracted both praise (Hansson et al. 2006; Lunshof et al. 2008; Sheehan 2011; Helgesson 2012; Kronenthal, Delaney, and Christman 2012) and criticism (Caulfield, Upshur, and Daar 2003; Hofmann 2009; Karlsen, Solbakk, and Holm 2011; Kaye 2012).

With biomedical big data, informed consent is likely to be rather unspecific too. Sensitive health-related data, such as genomic data for instance, are generally collected and made available through broad consent mechanisms. Moreover, for data collected beyond health services and research facilities, as is the case with the online space (e.g. social networking platforms, commercial apps, devices, or websites that allow patients to upload their data directly), information about data exposure is notoriously vague and hard to access. Furthermore, consent procedures often fall short of adequately informing data subjects about the terms of use of their data (Vayena, Mastroianni, and Kahn 2013). In such a scenario, people may experience a substantial lack of control over the flow of their data.

Another indirect means of data control for patients and research participants is the reliance on norms of professional confidentiality. Confidentiality is a professional’s capacity to keep a promise (often an implicit one) regarding the conditions of exposure of personal data. In the case of medical confidentiality, health information provided or acquired during medical or research activities will not be disclosed to third parties, unless this is required in order to fulfil the aims for which that information was originally collected. Confidentiality, therefore, assigns health data to a specific regime of (generally very limited) exposure. It is often assumed that the more medical activities rely on data, the less likely it is that confidentiality will be respected (Lunshof et al. 2008; McGuire and Gibbs 2006). The need to circulate data for research purposes may thus conflict with data subjects’ expectations. As empirical studies show, this is especially the case with respect to data collected for healthcare purposes (Carman and Britten 1995; Sankar et al. 2003). Notably, a large part of biomedical big data will be constituted precisely by data acquired during ordinary health-related activities and converging in individual electronic health records.

Another indirect way of respecting individual subjects’ control over their personal data has typically been anonymization. In its canonical form, anonymization renders a data set non-identifiable by removing personal identifiers (full anonymization) or by replacing them with codes or keys that the original data controller can use to re-identify the data upon necessity (pseudo-anonymization). Therefore, with anonymization, further uses of the data do not relate back to the person or her identity. Anonymization as a means of control, however, suffers from two limitations. The first is conceptual: anonymous use of data does not necessarily enable control over uses for specific purposes. For example, a person might not wish their data to be contributing to a particular kind of research due to social, cultural, religious, or other reasons. But individual control over the purpose of use cannot be exercised if the data have been anonymized. In this respect, anonymization may in fact hinder autonomy. This is particularly important to notice since anonymization often is portrayed as an effective mechanism to guarantee both privacy and autonomy—a move that can lead to confusion of two closely related but distinct values with different demands. The second limitation is pragmatic: there is mounting evidence pointing to the actual weaknesses of anonymization technologies, (McGuire and Gibbs 2006; Sankararaman et al. 2009; Visscher and Hill 2009; Gymrek et al. 2013) through increasing capabilities in data analytics. Hence, thinking about anonymization as if it were absolutely reliable creates a false sense of security and ultimately a false sense of control.

This brief overview shows that available means of both direct and indirect data control may be unable to meet people’s expectations with respect to their biomedical big data. Therefore, we now turn to exploring novel models of control that may help overcome the challenges highlighted so far.

## 4. Emerging Models of Control over Health Data

The shortcomings of the above-mentioned models of control are reflected in public concerns, as seen in data cited earlier in this paper. Given the value that people seem to attribute to control over personal data, it is not surprising that data-centred activities are met with disquiet. Currently, most of us do not really know how our personal data are being used nor who has access to them (Solove 2006; Brunton and Nissenbaum 2016). Yet people continue to make their data available to a multitude of operators and service providers—under the possibly misled assumption that actual threats to their privacy and interests are in fact negligible (Haeusermann et al. 2017). Moreover, pushing companies and institutions to openly communicate what they do with the data they have about consumers and citizens can be far from trivial (Turilli and Floridi 2009). Furthermore, even having one’s personal data corrected, changed, or deleted can be cumbersome once that data are present in a database and shared with third parties (Bennett 2012). The disconnect between the value of control on personal data and its increasing loss in the new constellations of powers of the data ecosystem, has given rise to several attempts aiming at supporting individuals to gain more control over their personal data.

**Fig. 1:**
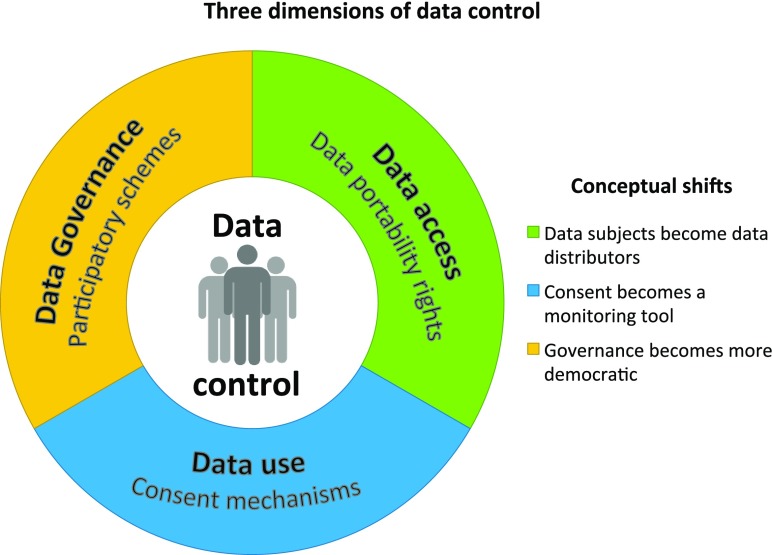
Three dimensions of data control

For analytical purposes, we can unpack the notion of control along three dimensions that debates on data protection in biomedical research consistently highlight: control over data access, control over data uses, and finally, control through governance (Fig. 1). These layers are interconnected as control in one dimension (e.g. over data access) may have implications on other dimensions too (e.g. data use). Moreover, we take each of the three dimensions to be a promising but not sufficient condition for control over biomedical big data. As we will see, not all of them bring about exclusively individual forms of control.

The first dimension of data control—that is, control over data access—refers to one’s ability to filter access to health data or personal health information by organizations or people. Access control is a fundamental dimension of control because it is immediately material to the conditions of exposure of that information. The second dimension of data control—over *data uses*—is also important since, other than deciding on who gets access to data, data control also requires the capacity to understand what one’s data are being used for and to decide whether those uses resonate with or are contrary to one’s interests and expectations. Data access and control have been considered particularly relevant in the case of genetic data (Rothstein 1997, 455). With biomedical big data, however, exerting those prerogatives might prove cumbersome. For this reason, new models of control over data access and use are needed to promote data subjects’ interests in this area. The third dimension of control over data has to do with *governance structures*, and has received a lot of attention, especially in the fields of genetic and genomic research and biobanking. In these areas, the vision has matured that designing governance structures, in which patients and participants can have their voices heard, will overcome the shortcomings caused by unspecific consent, thus fostering trust and accountability (O’Doherty et al. 2011).

### 4.1 Control Over Data Access and Novel Rights

Data protection legislation defines and promotes data subjects’ rights and interests with respect to personal information. In particular, data protection laws can regulate under which conditions access to personal information is possible and who has the power to limit or to grant access to personal data. In the United States, only some sectors possess specific data protection legislation. Nonetheless, data subjects in the United States are protected by a bundle of rights (such as the right to notice and to consent) concerning access to their data (Solove 2012). For instance, in the field of healthcare, the Health Insurance Portability and Accountability Act (HIPAA), enacted in 1996, sets specific restrictions on specific actors involved in processing health-related data. The European Union, instead, has had a directive on data protection since 1995.^3^ This directive has been replaced by the General Data Protection Regulation (GDPR) (EU 2016/679) released by the European Parliament and the Council in 2016 (and to be implemented in 2018) which introduces a number of individual entitlements on personal data (EU 2016/679). Of particular interest for our present discussion is the right to data portability. Article 20(1) of the GDPR affirms that

… [the] data subject shall have the right to *receive the personal data concerning him or her*, which he or she has provided to a controller, in a structured, commonly used and machine-readable format and have the right *to transmit* those data to another controller without hindrance from the controller to which the personal data have been provided. (emphasis added)

Interestingly, according to the GDPR, whereas for other data subjects’ rights European or Member States’ law can create derogations in the case of scientific research (art. 89.2), data subjects retain portability rights even if their data is being processed for research purposes.

This requirement will allow patients to ask for a copy of their electronic health records or to require data from a medical device. Furthermore, data portability will allow patients to ask for the transfer of such data to another entity. Companies, research institutes, and healthcare providers will have to enable this right without posing technical obstacles. Such requirement will thus foster data interoperability, competition, and data accessibility. But most importantly, this right goes beyond the right to access or to rectify one’s data. Through data portability, a data subject might make data collected by one research institution accessible to another. Or she could make data from a commercial application or device—such as an activity tracking system—available for health-related research. Moreover, at least in the GDPR, the article on data portability is distinct from the right to erasure (also commonly known as “the right to be forgotten”). This right gives individuals the option to request the deletion of their personal data if there is no compelling reason for their continued processing. The “right to be forgotten,” which generated significant debate in Europe and elsewhere, exempts data that are processed for public health purposes or scientific research. It therefore does not necessarily apply to health data (strictly defined). With a view to our earlier point about the expanded sources of health data, however, it will have substantial implications for health research purposes, Nevertheless, it should be pointed out that the right to data erasure may present practical limitations in all those cases in which personal data have been copied, divided in smaller “data-packages,” and distributed to multiple other parties for a variety of uses. In those circumstances, implementing the right to be forgotten will arguably be hindered by the difficulty of tracing precisely where data have been distributed. This problem is especially acute in the case of anonymized data. Such limitations, however, should not undermine data subjects’ rights but rather promote more efficient data management practices.

What data portability and the right to erasure realize is thus a conceptual shift whereby data subjects acquire a form of control that they previously did not have. In particular, thanks to data portability, data subjects acquire the role of data distribution hubs—a role previously reserved for data controllers. The case of data portability illustrates that an epochal increase in the amount of personal data does not automatically correspond to a decrease in individual control. Legislation, at least in this case, offered an innovative move in constructing a novel form of control over access to data. The implementation of the GDPR and specifically the operationalization of the data portability right will reveal whether such a move can actually achieve its multiple aims. Notably, it remains to be seen whether procedures will be sufficiently streamlined to ensure that data portability rights will indeed be widely enjoyed. Nevertheless, it remains a novel approach that is currently also under consideration beyond Europe (MacGillivray and Shambaugh 2016).

### 4.2 Control Over Data Uses and Novel Consent Models

With the unparalleled growth of information and communication technologies, for the last two decades most human activities have been migrating online (Floridi 2015). Activities that used to happen on paper are now commonly undertaken with the intermediation of digital tools. This is also starting to be the case for informed consent. In December 2016, the U.S. Department of Health and Human Services (DHHS) and the Food and Drug Administration (FDA) released specific—albeit nonbinding—guidance for the use of electronic informed consent (FDA and DHHS 2016). According to these guidelines electronic informed consent (eIC)

… may be used to provide information usually contained within the written informed consent document, evaluate the subject’s comprehension of the information presented, and document the consent of the subject or the subject’s [legally authorized representative]. Electronic processes to obtain informed consent may use an interactive interface, which may facilitate the subject’s ability to retain and comprehend the information. Furthermore, these electronic processes may allow for rapid notification to the subjects of any amendments pertaining to the informed consent that may affect their willingness to continue to participate. (FDA and DHHS 2016, 3)

One major advantage of eIC is the exploitation of electronic design to enhance information disclosure and therefore to promote a better understanding of the *conditions of use* of a person’s data. The electronic processing of information may help streamline participants’ choices as to whether current and envisaged data uses resonate with their expectations. In turn, this can result in an increased level of control over the use of one’s data.

Especially relevant to data disseminated in online environments is the development of electronic consent management mechanisms (ECMMs) (Bonnici and Coles-Kemp 2010; Grady et al. 2017). Online, where informed consent of the kind used in healthcare and research is not required, ECMMs are expected to increase trust in operators and to satisfy users’ online privacy preferences (Grady et al. 2017).

Building on such approaches, organizations like Sage Bionetworks have developed the Participant-Centered Consent (PCC) toolkit, an ECMM that is suitable to research activities and indeed promising in particular for biomedical big data research (Sage Bionetworks 2017). The PCC toolkit is intended for all research groups planning to initiate a study involving human research subjects and is especially suited for mobile app-mediated research studies. Paper-based informed consent procedures have long been facing harsh criticism for being ineffective in conveying understandable and relevant information to enable participants’ voluntary choices (Manson and O’Neill 2007). The aim of PCC is precisely to overcome these limits and to “maximize [participants’] informedness, comprehension and voluntariness” (Sage Bionetworks 2017, ¶5). The toolkit draws on recent advances in user experience design and consists of a variety of open source visual resources (like icons and animations), templates, and electronic consent workflows that researchers can use for enroling participants in their studies. Patient-centred designs for eIC shall then be approved by Institutional Review Boards (IRBs) before being used—a step that Sage Bionetworks recommends also for human subject research by non-research institutions and companies, which, in some jurisdictions, are not obliged to seek IRB approval. Moreover, PCC is not limited to the online environment, as it can also be used in the context of more conventional face-to-face enrolment procedures. Enabling greater understanding of the conditions of use of health data and personal health information, PCC utilizes design to help improve data subjects’ control over their data.

Other innovative forms of consent that aspire to improve individual control include the dynamic consent model (Kaye et al. 2015; Budin-Ljøsne et al. 2017). Building on the model of tiered consent (McGuire and Beskow 2010; Mello and Wolf 2010; Bunnik, Janssens, and Schermer 2013) the dynamic consent model aims at embedding evolving data subject’ preferences into an open communication process between participants and researchers. The latter, as part of the initial informed consent process, are supposed to ask participants which uses of their data they allow, and which, instead, they do not consent to (in the same way as tiered consent does). When data is used and reused for different research projects, they are only used according to the indicated preferences of participants, who are notified of such use. Participants are able to change their preferences regarding data uses throughout the duration of the project. Dynamic consent, in other words, turns consent from a static to an adaptive process, thus increasing participants’ control over the use of their data.

It is worth noting that innovative consent models to promote more granular control over health data and personal health information may have an impact on the quality of science. In particular, widespread opting-out may bias research datasets and compromise the reliability and reproducibility of data analyses in specific domains of science. This is certainly a serious matter. Some have even argued that contributing to medical research (through direct participation in interventional clinical trials) is indeed a reciprocity-based moral obligation (Harris 2005). The same argument could be extended to the provision of health data and personal health information for research purposes. Analysing the ethical foundations of such a line of argument is beyond the scope of the present paper. Yet we concede that control is not absolute, in the sense that it can be waived when it conflicts with other valuable states or outcomes—public health, national security, and criminal investigations being typical cases, in which it can be acceptable to bypass individuals’ entitlements to informational control. However, as a rule, in order to trump an individual entitlement to controlling personal health information, there should be solid evidence supporting a causal link between the existence of control rights and the decreasing quality of research datasets.

Also of relevance to the present discussion, there is growing interest in exploring the feasibility of financial transaction models for data acquisition. The business model of some genomic testing companies is based on offering financial rewards to individuals who provide access to their data (Roberts, Pereira, and McGuire 2017). Moreover, patients have claimed that a fair data market should also reward them (Farr 2016). We are still in the early days of this discussion, but it is likely that such models might impact data subject’s control of data uses. It remains to be seen how such transaction-based models may work, if at all, as they certainly require a major break from the current altruism-based framing of the common good of research. It is also questionable whether a *quid pro quo* approach in this area would increase individual control or rather give more control of data to corporations that could possibly manipulate data markets.

As in the case of data portability rights, the models above are also representing a conceptual shift. Thanks to innovations such as eIC, PCC, and dynamic consent, some of the shortcomings of the classic consent in, for instance, effective disclosure or respecting changing preferences of participants can be overcome. Therefore, while consent innovation is not a panacea to respecting choice and enhancing control, it is certainly an area that holds considerable promise. As to data transaction models, should they be implemented, they would surely again illustrate changes in concept that go beyond mere changes in process.

### 4.3 Augmented Control Through Participatory Governance Schemes

The growth of research activities based on large collections of human biological material has been spectacular in the last two decades (Hirtzlin et al. 2003). At the same time, thanks to the ever-declining cost of genome sequencing, interest in the analysis and clinical use of large human DNA collections has grown consistently around the world (Berg, Khoury, and Evans 2011). Those tendencies have turned human biological resources and data into extremely precious assets for health-related research. In order to increase access to those resources, a series of regulatory changes has taken place, especially, in the process of obtaining consent from samples and data donors. It has been argued, however, that with the growth of large biobanks and human data repositories, innovation should occur also at the level of the governance mechanisms for such research infrastructures (O’Doherty et al. 2011). In the field of biomedical big data, we reckon, following such injunction would not only create more accountability but also increase the degree of control that data subjects can exert on the data they make available for research. This resonates with narratives mobilized in the field of citizen science (Irwin 1995; Silvertown 2009) and participant-led medical research (Vayena and Tasioulas 2013). Such initiatives enable data donors to make direct use of the data resources they contribute to create. As biomedicine ventures into the world of big data, health relevant data will increasingly be produced and contributed directly by patients or healthy individuals. This prefigures the possibility that, in biomedical big data research, citizen science models will offer data subjects additional means of control over the conditions of exposure of their data.

Whether subjects’ control will increase depends also and possibly to a greater extent, on the governance mechanisms of biomedical big data research. Indeed, novel approaches are emerging in this field that blend features of citizen science models and participatory arrangements of data governance. For instance, the data cooperative model pioneered by MIDATA.coop (https://www.midata.coop/) represents an example of how data subjects can acquire control over their data through governance mechanisms of a novel type (Hafen, Kossmann, and Brand 2014; Hafen 2015). The aim of MIDATA is to store health relevant data from a variety of sources and to make them available for research projects, enabling at the same time data subjects to decide about the use of their data. MIDATA has a cooperative structure, whereby all data contributors are also owners of the collection. MIDATA is a not-for-profit organization, but revenue potentially generated through users’ will be re-invested in the maintenance of the cooperative or to fund research. Most importantly, the cooperative has an oversight mechanism through which the partners get to review all data access requests and decide collectively whether to grant or deny access to their data. One of the components of this oversight mechanism is an ethical reference framework (including adapted informed consent forms and specific principles) that should support the decisional processes on data access and sharing. MIDATA aims to develop a network of regional cooperatives, potentially anywhere in the world, and to offer open source software to develop data analytics. In this way the notion that data subjects should be directly in control of their data can be swiftly applied to different national contexts as well as to international research projects primed on the analysis of datasets from different countries.

In this model, control over data exposure is thus enabled by a governance tool—one that is a logical consequence of the cooperative principle that MIDATA is based upon. The kind of control is of a collective type, since data subjects decide together on access to the collection as a whole.

MIDATA has currently been adopted by two scientific research projects, one on multiple sclerosis (including data generated from physicians, patients, and portable devices) and one on the effects of bariatric surgery (drawing on data generated by patients through the use of smart scales, a step counter, and a dedicated app).

Interestingly, the MIDATA model embraces a narrative of partnership and meaningful engagement that is also emerging in other areas such as in the field of precision medicine (Blasimme and Vayena 2016, 2017). This instance of governance-based control, produces a conceptual shift in the notion of participation itself, in particular, by framing the issue of data governance as one of data democracy. As a consequence, research participants are cast as a community that has interests and entitlements in controlling its data.

However, a potential limitation of emerging forms of data democracy needs to be highlighted. Active participation in data democracy platforms requires certain skills and a given level of awareness. As a consequence, it may well be the case that individuals taking more active roles in science governance through data democracy models belong to socially and culturally homogeneous strata of the population. Moreover, it is also possible that certain disease groups—on the basis of their strong motivation—acquire leading roles in these platforms, allowing their voice to be heard more prominently than that of other legitimate stakeholders. Should that be the case, data democracy initiatives will have to actively seek to redress such imbalances by removing barriers to participation and by reaching out to include more diverse players.

## 5. Conclusion

We have argued that the capacity to exert control over the conditions of exposure of health data and personal health information data is ethically valuable. We began by spelling out the importance of control in the context of a broader network of values that control can be instrumental to. This constitutes a *prima facie* justification for saying that control, whenever possible, should be on offer to data subjects. Contradictory as it might sound, ceding control is one of the possible ways of exercising it.

Empirical evidence shows that people are concerned at the prospect of losing control over their data and, as we showed, they have some good moral reasons for being concerned. In the context of biomedical big data research, the issue of control and its associated risks has become prominent. As medical research moves towards the use of ever more diverse types of data, accepting loss of informational control is not necessarily the only option.

Our examples show that individual control can actually be promoted through innovative models of data access, use, and governance. Moreover, improving control in any of these three areas is likely to have an impact on the others. The synergistic effect of those novel models will therefore result in more opportunities to control health data and personal health information, at least for data subjects who are interested in doing so. This will not only serve some important moral interests of those individuals. It will also help building trust around data-driven medical paradigms and flagship research initiatives—such as clinical genomics, precision medicine, and digital health—both in the public and in the private sector. Failure to disclose the collection and destination of health data gathered by general practitioners—as in the case of care.data in the United Kingdom (Triggle 2014)—or the transfer of data from the public healthcare system to a private company—as recently happened between the British National Health Service and Google’s DeepMind (Powles and Hodson 2017)—led to public controversies. Arguably, at the heart of such controversies lies not the utility of collecting and analysing patients’ data but the ways in which those initiatives bypassed individuals’ control over their data. This attitude shows insufficient consideration for the ethical value of control and, for this reason, elicited numerous reactions. Attributing to control the ethical consideration it deserves—albeit insufficient to account for all the ethical aspects of biomedical big data—is indispensable to respond to data donors’ legitimate expectations, as well as to cultivate a climate of public trust with respect to data-driven medical research.

## Footnotes

1 The Eurobarometer is a wide-ranging series of public opinion surveys conducted regularly on behalf of the European Commission in the twenty-eight member states of the European Union (European Commission 2015).

2 Our definition of control owes to Alan Westin’s influential definition of privacy as “the claim of individuals, groups, or institutions to determine for themselves when, how, and to what extent information about them is communicated to others” (Westin 1967, 7). However, as we explain in this section, we do not see control only as a function of privacy, nor we endorse Westin’s notion that privacy is mainly about informational control.

3 A right to privacy has nonetheless been present in the European law since the promulgation of the European Convention on Human Rights (art. 8), signed in Rome in 1950.
